# Efficacy and Safety of Computed Tomography-Guided Percutaneous Balloon Compression under Local Anesthesia for Recurrent Trigeminal Neuralgia: A Prospective Study

**DOI:** 10.1155/2024/8885274

**Published:** 2024-04-09

**Authors:** Lulu Xi, Xiaohui Liu, Hongchen Shi, Wenbiao Han, Liqin Gao, Li Wang, Junpeng Liu, Yue Ren, Yuanyuan Du, Guangzhao Liu

**Affiliations:** ^1^Department of Pain, Second Hospital of Hebei Medical University, 215 Hepingxi Road, Shijiazhuang, Hebei 050000, China; ^2^Department of Neurology, Second Hospital of Hebei Medical University, 215 Hepingxi Road, Shijiazhuang, Hebei 050000, China

## Abstract

**Purpose:**

There are several ways to treat trigeminal neuralgia (TN); however, TN may recur after treatment. This study investigated the efficacy and safety of computed tomography (CT)-guided percutaneous balloon compression (PBC) under local anesthesia for treatment of recurrent trigeminal neuralgia. *Patients and Methods*. This is a prospective and nonrandomized controlled clinical study. Forty-eight patients with classical TN were scheduled to undergo PBC surgery at the pain department of our institution between January 2021 and June 2021. The patients were prospectively divided into an initial onset group, A (21 cases), and a recurrence group, B (27 cases). All surgeries were performed with CT guidance and under local anesthesia. Postoperative complications were also observed. Pain was assessed using the visual analog scale (VAS) and Barrow Neurological Institute (BNI) scale. Efficacy indices were evaluated at 3, 6, 12, and 18 months after surgery.

**Results:**

All participants reported complete pain relief at discharge. After 18 months of follow-up, the total effective rate of pain control was 89.5% (group A, 90.5%; group B, 88.8%). There was no significant difference in the BNI scores between the two groups before and after treatment. All patients had hypoesthesia on the affected side, and no severe complications such as diplopia, blindness, intracranial hemorrhage, or intracranial infection occurred.

**Conclusions:**

CT-guided PBC under local anesthesia is safe and effective for the treatment of recurrent TN and thus acts as an effective alternative for geriatric patients and those with high-risk factors.

## 1. Introduction

Trigeminal neuralgia (TN) is one of the most painful diseases, characterized by recurrent, unilateral, transient but severe, electrocution like pain with rapid onset and short duration (up to several minutes) [1]. It can occur when talking, washing, or eating, followed by sudden cessation of pain and reappearance after the next stimulus. The patient's quality of life is severely reduced. According to the International Classification of Headache Disorders 3rd edition (ICHD-3), TN can be classified into three subgroups: classical TN, secondary TN, and idiopathic TN [2]. Several treatments for classical TN are available and have been repeatedly reported for patients with poor pain control [3, 4], but these treatments only briefly manage the pain and do not prevent recurrence. Microvascular decompression (MVD) is currently considered to provide the lowest recurrence rate; however, following recurrence, patients are often reluctant to undergo another craniotomy, and MVD is not suitable for older patients or those with poor health. Therefore, PBC can replace microvascular decompression in older patients [5].

Percutaneous balloon compression (PBC) has been shown to be effective in treating TN. In 1983, Mullan and Lichtor performed PBC for the first time to treat TN [6]. Efficacy and safety of awake computed tomography (CT)-guided PBC of the trigeminal ganglion for the treatment of TN has been reported [7, 8]. Its advantages include sufficient nerve damage, no need to distinguish the responsible nerve, and simple and safe operation. When the balloon catheter enters the Meckel's cavity, the trigeminal nerve semilunar ganglion is compressed by the balloon for approximately 1–3 minutes. The balloon is then released, and the balloon catheter is removed. After the trigeminal ganglion is damaged by compression, an analgesic effect is achieved in the innervated area.

PBC is usually performed under general anesthesia and C-arm guidance, which poses a great risk to patients with respiratory or cardiovascular complications and a high incidence of trigeminal cardiac reflex (TCR). Trigeminal ganglion block-assisted deep sedation can reduce the occurrence of serious complications [9]. This study was performed under local anesthesia. By comparing PBC in the treatment of primary and recurrent TN, we explored the efficacy and safety of CT-guided PBC under local anesthesia for the treatment of recurrent trigeminal neuralgia.

## 2. Methods

### 2.1. Study Design and Participants' Population

**Figure 1:**
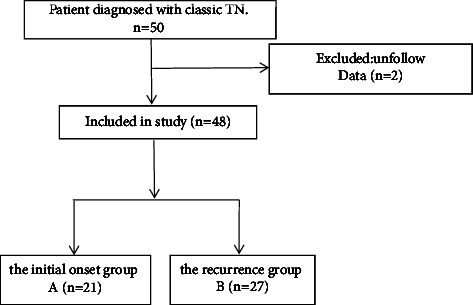
Flowchart of inclusion of trigeminal neuralgia patients.

This is a prospective and nonrandomized controlled clinical study. From January 2021 to June 2021, we enrolled 48 patients with TN in the pain department of our institution. All patients were diagnosed with classical TN, with one or more affected trigeminal branches. The study was conducted in accordance with the Declaration of Helsinki and approved by the Ethics Committee of our institution (2020-R562). Written consent was obtained from all participants in this study. All surgeries were performed by the same surgeon, and the medical records of all patients were collected and analyzed. The patients were divided into an initial onset group, A (21 cases), and a recurrence group, B (27 cases). A flowchart of the entry and exit of patients is shown in Figure 1. All surgeries were performed with CT guidance and under local anesthesia. The inclusion criteria were as follows: (1) definite diagnosis of classical TN; (2) age between 30 and 90 years; and (3) a visual analog scale (VAS) score ≥4 points. On the other hand, the exclusion criteria were as follows: (1) age <30 years or >90 years; (2) local infection at the puncture site; (3) coagulopathy or hemorrhagic disease; (4) mental illness preventing cooperation; and (5) severe heart, brain, lung, and liver diseases.

### 2.2. Surgery

**Figure 2:**
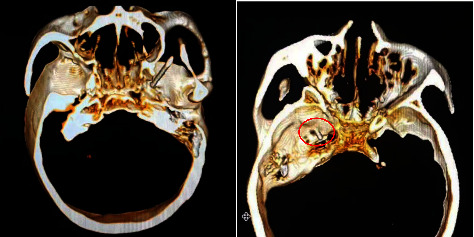
If the foramen ovale is small, it will cause difficulty in piercing. Ct-guided needle puncture significantly improved the accuracy. The injection of anesthetics into Meckel's cavity should be avoided during local anesthesia, and the 3D digital imaging technology can clarify the tip location and improve the safety.

**Figure 3:**
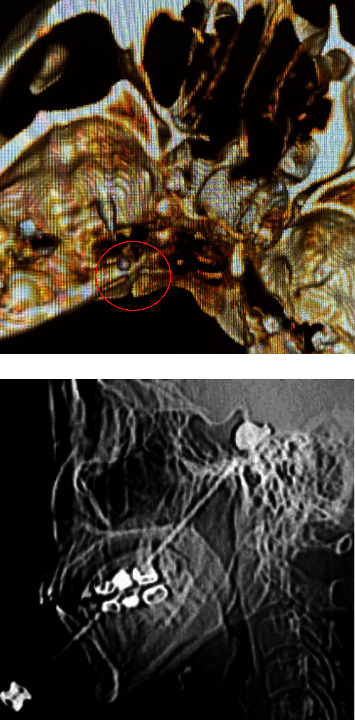
The location of the balloon catheter can be determined by inserting the balloon catheter under CT guidance. After the iohexol was injected, a “pear-shaped image” was displayed on lateral CT imaging, indicating successful surgery.

The procedure of surgery has been described previously [10]. The patient remained awake during the operation and was placed in the supine position with the head tilted backward. ECG monitoring was routinely performed to monitor blood pressure, heart rate, and oxygen saturation. Midazolam 0.03 mg/kg was slowly injected intravenously for sedation before surgery. A 22G local anesthesia needle with a length of 10 cm was used for the puncture. Under CT guidance, 1% lidocaine (0.5 mL) was injected into the foramen ovale of the trigeminal nerve ganglia to block the nerves (Figure 2). The guidewire was then placed into the local anesthesia needle, the local anesthesia needle was withdrawn, and a 14G balloon puncture needle was placed along the guidewire. When the needle was advanced into the foramen ovale, a No. 4 Fogarty balloon catheter was inserted along the balloon catheter needle. After confirming the proper location, 0.5–0.8 ml iohexol was injected into the catheter to inflate the end balloon. During the CT scan of the lateral head, the balloon and position were adjusted until a pear-shaped image was obtained (Figure 3). After 1.5–2.5 min of compression (group A, 1.5 min; group B, 2.5 min), the balloon was withdrawn.

### 2.3. Therapeutic Assessment and Follow-Ups

The Barrow Neurological Institute (BNI) scale and visual analog scale (VAS) were used to assess the degree of pain. Complications and pain recurrences were also recorded. The BNI scale assessed the pain intensity in patients, as shown in Table 1. A BNI Pain Intensity Score of I-II and a VAS score of <3 were considered effective. Patients were followed up for 3, 6, 12, and 18 months after surgery, and their pain and numbness were recorded.

### 2.4. Statistical Analysis

SPSS 24 software and GraphPad Prism 8 were used for statistical analyses. After analysis, all variables were normally distributed. The sample size was calculated using the G^*∗*^power software (Heinrich-Heine-University Düsseldorf, version 3.1.9.4, Düsseldorf, Germany). We assumed that the effect size for the main outcome of the effective rate was 0.45, the alpha error at 0.05, and the power at 0.80, considering 20% dropout rate; thus, 48 participants were recruited in the study. The data were presented as mean ± standard deviation. Categorical variables were analyzed using *χ*^2^ test or Fisher exact probability test, and continuous variables were analyzed using variance analysis of repeated measurement data. *P* < 0.05 was considered to be statistically significant.

## 3. Results

### 3.1. General Demographics

This is a prospective and nonrandomized controlled clinical trial study. There were 48 patients (20 males and 28 females). Ages ranged from 35 to 89 years (mean 61.81 ± 13.38 years). The disease duration ranged from 0.5 to 420 months. Seventeen patients had left-sided pain, 29 had right-sided pain, and two had bilateral pain. Among them, there were three cases of V1, four cases of V2, 15 cases of V3, 11 cases of V1 + V2, one case of V1 + V3, 13 cases of V2 + V3, and one case of V1 + V2 + V3. Before treatment in the pain department, three of the 27 patients with recurrence underwent MVD, and five underwent pulsed radiofrequency. Percutaneous radiofrequency thermocoagulation (PRT) and PBC were performed in 18 patients, and gamma knife radiosurgery (GKRS) was performed in 1 patient. General demographic data are shown in Table 2.

### 3.2. Clinical Outcome

There were 21 patients in group A with initial trigeminal neuralgia and 27 patients in group B with recurrent trigeminal neuralgia. There were no significant differences in sex (*P*=0.942), age (*P*=0.107), VAS scores (*P*=0.643), or BNI scores (*P*=0.777) between groups A and B. The blood pressure and heart rate remained stable. All patients underwent microballoon compression and dilation of the trigeminal nerve; five patients still had postoperative pain, which disappeared 7 days after repeated surgery.

**Figure 4:**
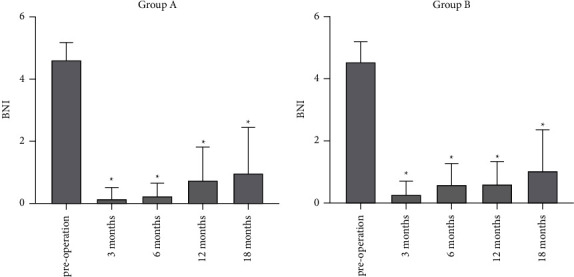
Changes in BNI scores after percutaneous balloon compression compared with preoperative scores, ^*∗*^*P* < 0.05. BNI, Barrow Neurological Institute.

According to the BNI, the rate of pain relief (BNI I-II) within 6 months in groups A and B reached 100%. In group A, the pain recurred in one case (4.7%) within 12 months; in group B, the pain recurred in two cases (7.4%), *P*=0.595. In group A, pain recurrence occurred in two cases (9.5%); in group B, pain recurrence occurred in three cases (11.1%) within 18 months, *P*=0.621. 2. The postoperative BNI scores in groups A and B were significantly improved compared to those before surgery, as shown in Figure 4.

After 18 months of follow-up, the total effective rate of pain control was 89.5% (group A, 88.8%; group B, 90.4%, *P*=0.612). The mean VAS score of group A was 6.48 ± 0.92 before treatment, 0.29 ± 0.56 after 3 months, 0.67 ± 0.73 after 6 months, 1.10 ± 1.33 after 12 months, and 1.43 ± 1.32 after 18 months. The mean VAS score of group B was 6.59 ± 0.79 before treatment, 0.44 ± 0.69 after 3 months, 0.74 ± 0.81 after 6 months, 1.22 ± 1.25 after 12 months, and 1.42 ± 1.52 after 18 months. There was no significant difference in VAS scores between groups A and B during the follow-up period. The VAS scores are presented in Table 3. There was no significant difference in BNI and VAS between the two groups at each time point. VAS was compared between the two groups before and after the operation (before surgery, 95% CI of diff. −0.866∼0.580, *P*=0.989, 3 months after surgery, 95% CI of diff. −0.660∼0.374, *P*=0.954, 6 months after surgery, 95% CI of diff. −0.746∼0.556, *P*=0.997, 12 months after surgery, 95% CI of diff. −1.193∼0.907 *P*=0.998, and 18 months after surgery, 95% CI of diff. −0.865∼1.342 *P*=0.984). BNI was compared (before surgery, 95% CI of diff. −0.625∼0.434, *P*=0.993, 3 months after surgery, 95% CI of diff. −0.301∼0.397, *P*=0.998, 6 months after surgery, 95% CI of diff. −0.861∼0.385, *P*=0.842, 12 months after surgery, 95% CI of diff. −1.177∼0.415 *P*=0.677, and 18 months after surgery, 95% CI of diff. −1.096∼1.286, *P*=0.999).

### 3.3. Side Effects and Complications

All patients experienced postoperative hypoesthesia of the skin and mucosa on the affected side. Twelve patients experienced postoperative masticatory fatigue (three patients in group A, 14.2%; nine patients in group B, 33.3%, *P*=0.119). Within 8 h after surgery, 16 patients (7 patients in group A, 33.3%; 9 patients in group B, 33.3%, *P*=0.619) experienced headache; 7 patients (2 patients in group A, 9.5%; 5 patients in group B, 18.5%, *P*=0.327) experienced dizziness; and nausea or vomiting occurred in 17 patients (7 patients in group A, 33.3%; 10 patients in group B, 37.0%, *P*=0.517), all of which resolved spontaneously within 12 h and was considered to be caused by surgical stimulation. Two patients (group A did not present; 2 patients in group B, 7.4%, *P*=0.311) developed limited abduction of the affected eye with diplopia, and the symptoms disappeared within 6 hours after surgery. We considered the cause of this symptom to be the use of local anesthetics on the abductor nerve. Fourteen patients (6 patients in group A, 28.5%; 8 patients in group B, 29.6%, *P*=0.272) developed perioral and oral herpes simplex within 3 days after surgery and recovered within 2 weeks after oral administration of antiviral drugs. The PBC showed no keratitis, corneal numbness, other cerebrospinal fluid fistulas, cerebral nerve palsy, anesthesia pain, or intracranial hemorrhage during or after surgery. No deaths were reported. A comparison of complication data is presented in Table 4.

## 4. Discussion

TN is one of the most painful diseases. The prevalence rate of TN is from 4 up to 29 cases per 100,000 person-years [11]. Although the pathophysiological mechanism is not clear [12], various effective surgical treatments for TN have been applied, such as MVD, PBC, and radiofrequency thermocoagulation of the trigeminal semilunar ganglion. It has been reported that these treatments can achieve substantial curative effects but also have certain recurrence rates.

In terms of minimally invasive treatment of TN, the rate of pain relief after PBC was similar to that reported after PRT and higher than that of GKRS [13, 14]. PBC has a relatively low TN recurrence rate of approximately 20% within 5 years and 32% within 20 years, along with a patient-reported satisfaction rate of 70% [15, 16]. Thus, it could be preferentially recommended for patients undergoing ablative procedures [17]. Moreover, the technique provides a more stable and effective means of nerve injury without craniotomy and avoids the occurrence of serious complications to a large extent. The mechanism of PBC involves damaging the large and medium myelinated nerve fibers that transmit pain and preserving the unmyelinated nerve fibers that transmit to the corneal reflex [18].

General anesthesia can provide patients with a more comfortable feeling [19, 20]. However, when the balloon compresses the trigeminal ganglion, TCR often occurs, leading to a sharp drop in blood pressure and heart rate, and even causing cardiac arrest. Wang et al. reported that atropine pretreatment before compression of the trigeminal nerve ganglion was more reasonable in preventing significant hemodynamic changes [21]. We found in the clinic that the incidence of blood pressure drop in patients was still high even after preoperative preconditioning. Local anesthesia blocks TCR from occurring and maintains stable blood pressure and heart rate [22]. There was no significant difference in blood pressure and heart rate before and during surgery. The needle location was carefully monitored. If the tip is too deep, the anesthetic may be injected into the Meckel's cavity, causing serious consequences. This should be avoided and therefore precise positioning is required.

To date, CT-guided surgery is the best strategy for improving surgical safety. Three-dimensional (3D) imaging reconstruction produces more efficient and safer results than two-dimensional imaging [23]. This method has been widely used in PRT. However, for PBC, most of the surgery is still carried out under the C-arm. In our operations, we occasionally encounter anatomical anomalies such as skull deficit or the integration of the foramen ovale and the foramina spinosum. Under the C-arm, these conditions may be overlooked and serious consequences may occur intraoperatively. Preoperative 3D imaging reconstruction of the skull base is helpful in finding anatomical variations and avoiding inadvertent damage to the peripheral neurovascular structure [24]. The location of the foramen ovale and the depth of penetration of the cannula are visible, which gives the surgeon confidence and greatly improves patient safety. In addition, in patients with a bony protrusion around the foramen ovale, which prevents successful intubation using conventional techniques, 3D reconstruction is more effective and accurate than other techniques. Our operations were all completed under CT and are, therefore, different from traditional operations.

We have accumulated rich experience in PRT under local anesthesia and believe that PBC can also be completed under local anesthesia. Adequate anesthesia is of the utmost importance, and the injection site of the anesthetic drug must be ensured at the trigeminal ganglion. Pay attention to slow injection, repeatedly withdraw the syringe, to ensure that there is no blood and cerebrospinal fluid. During the operation, 1% lidocaine hydrochloride (0.5 mL) was injected into the trigeminal ganglion to inhibit TCR and improve safety. During the entire process, the patient was awake, and discomfort could be reported in a timely manner, which was convenient for the administration of the corresponding treatment and effectively avoided serious complications such as double vision and oculomotor nerve palsy. Local anesthesia surgery is more suitable when the patient is older or in poor health and cannot use general anesthesia.

Numbness is the most common complication of PBC. Studies have shown that the incidence of facial hypoesthesia after balloon compression is more than 90% [25]. Although patients in group B may have had symptoms of numbness as a result of damaging treatments such as radiofrequency thermocoagulation and glycerol injection, the numbness was significantly relieved when the pain recurred. After PBC, the range and degree of numbness increased significantly, which was also related to the location of PBC in the trigeminal ganglion. In patients with recurrence, we extended the compression time and intensity, which made the subjective feeling of postoperative numbness more obvious. Increased duration and degree of compression also increased the incidence of masticatory muscle weakness on the affected side. Twelve patients showed symptoms of masticatory weakness, all of whom recovered within 6 months.

After 18 months of follow-up, we compared the efficacy between groups A and B and found that the total effective rate of pain control was 89.5% (group A, 88.8%; group B, 90.4%). There was no statistically significant difference in the postoperative efficacy between the two groups. This suggests that treatment in both groups was safe and effective. The preoperative and postoperative VAS scores differed significantly between the two groups. All patients developed hypoesthesia in the skin and mucosa of the affected side immediately after surgery, presenting a primary complication. Temporary postoperative discomfort, such as headache, dizziness, and nausea or vomiting, was associated with irritation of the surrounding tissues or nerves during the surgical procedure. All the symptoms resolved after rest. No intracranial hemorrhage, intracranial infection, or other serious complications occurred in either group, indicating that PBC was safe and effective.

This was a single-center study with a small sample size and a short follow-up period of 18 months. The determination of the long-term efficacy of PBC in the treatment of postoperative recurrent TN requires a larger sample size, longer follow-up time, and multicenter evaluation.

## 5. Conclusion

CT-guided PBC under local anesthesia is safe and effective for treating recurrent TN. This approach provides a safe and effective alternative for older patients and those with high-risk factors.

## Figures and Tables

**Table 1:** Barrow Neurological Institute (BNI) pain intensity scale.

Score	Description
I	No pain, no medication
II	Occasional pain, not requiring medication
III	Some pain, adequately controlled with medication
IV	Some pain, not adequately controlled with medication
V	Severe pain, no pain relief

**Table 2:** Patient demographics and clinical data.

Characteristics	Baseline (mean ± SD)
Gender (*n*)	
Male	20
Female	28
Age year	
Mean ± SD	61.81 ± 13.38
Range	35–89
Pain duration, month	
Range	0.5–420
Pain side (*n*)	
Right	19
Left	17
Both	2
Branches affected (*n*)	
V1	3
V2	4
V3	15
V1 + v2	11
V1 + V3	1
V2 + V3	13
V1 + V2 + V3	1
Preoperative VAS	Group A: 6.48 ± 0.92 Group B: 6.59 ± 0.79
Type of prior procedure (*n*)	
MVD	3
PRF	5
GKRS	1
Destructive procedures	18

VAS, visual analogue scale; MVD, microvascular decompression; PRF, pulsed radiofrequency; GKRS, gamma knife radiosurgery.

**Table 3:** Comparison of VAS score in the two groups.

	3 months	6 months	12 months	18 months
Group A	0.29 ± 0.56	0.67 ± 0.73	1.10 ± 1.33	1.43 ± 1.32
Group B	0.44 ± 0.69	0.74 ± 0.81	1.22 ± 1.25	1.42 ± 1.52

**Table 4:** Comparison of complications between the two groups.

Complications	Percentage (%)
Facial hypoesthesia	
Group A	100
Group B	100
Mastication weakness	
Group A	14.2
Group B	33.3
Facial herpes outbreak	
Group A	28.5
Group B	29.6
Diplopia (recovered in six hours)	
Group A	None
Group B	7.40
Headache (recovery within 12 hours)	
Group A	33.3
Group B	33.3
Nausea or vomiting (recovery within 12 hours)	
Group A	33.3
Group B	37.0
Dizziness (recovery within 12 hours)	
Group A	9.5
Group B	18.5

## Data Availability

The datasets generated during and/or analyzed during the current study are available from the corresponding author upon reasonable request.
